# Text-mining assisted regulatory annotation

**DOI:** 10.1186/gb-2008-9-2-r31

**Published:** 2008-02-13

**Authors:** Stein Aerts, Maximilian Haeussler, Steven van Vooren, Obi L Griffith, Paco Hulpiau, Steven JM Jones, Stephen B Montgomery, Casey M Bergman, The Open Regulatory Annotation Consortium

**Affiliations:** 1 Laboratory of Neurogenetics, Department of Molecular and Developmental Genetics, VIB, Leuven, B-3000, Belgium; 2 Department of Human Genetics, Katholieke Universiteit Leuven School of Medicine, Herestraat, Leuven, B-3000, Belgium; 3 Institut de Neurosciences A Fessard, Centre National de la Rechere Scientifique, Gif-sur-Yvette, 91 198, France; 4 Department of Electrical Engineering, Katholieke Universiteit Leuven, Heverlee, B-3001, Belgium; 5 Canada's Michael Smith Genome Sciences Centre, British Columbia Cancer Agency, Vancouver, V5Z 4E6, Canada; 6 VIB Department for Molecular Biomedical Research, Ghent University, Ghent, 9052, Belgium; 7 Wellcome Trust Sanger Institute, Hinxton, CB10 1SA, UK; 8 Faculty of Life Sciences, University of Manchester, Oxford Road, Manchester, M13 9PT, UK

## Abstract

**Background:**

Decoding transcriptional regulatory networks and the genomic *cis*-regulatory logic implemented in their control nodes is a fundamental challenge in genome biology. High-throughput computational and experimental analyses of regulatory networks and sequences rely heavily on positive control data from prior small-scale experiments, but the vast majority of previously discovered regulatory data remains locked in the biomedical literature.

**Results:**

We develop text-mining strategies to identify relevant publications and extract sequence information to assist the regulatory annotation process. Using a vector space model to identify Medline abstracts from papers likely to have high *cis*-regulatory content, we demonstrate that document relevance ranking can assist the curation of transcriptional regulatory networks and estimate that, minimally, 30,000 papers harbor unannotated *cis*-regulatory data. In addition, we show that DNA sequences can be extracted from primary text with high *cis*-regulatory content and mapped to genome sequences as a means of identifying the location, organism and target gene information that is critical to the *cis*-regulatory annotation process.

**Conclusion:**

Our results demonstrate that text-mining technologies can be successfully integrated with genome annotation systems, thereby increasing the availability of annotated *cis*-regulatory data needed to catalyze advances in the field of gene regulation.

## Abstract 2

Text-mining technologies can be integrated with genome annotation systems, increasing the availability of annotated *cis*-regulatory data.

## Background

The process of annotation is an essential first step in attributing biological information to genome sequences. Traditionally, the main focus of genome annotation has been the identification and annotation of well-studied biological entities, such as protein-coding genes, RNA genes and repetitive DNA. Efforts to annotate these genomic features typically adopt one of several established annotation paradigms - the 'museum,' 'jamboree,' 'cottage industry,' or 'factory' models of genome annotation (reviewed in [1,2]). Other important functional regions of genomes that are more difficult to predict by *ab initio *or homology methods are often omitted from the standard genome annotation process, in particular the *cis*-regulatory sequences that control transcription. Instead, *cis*-regulatory sequences are typically annotated by manual curation from the literature either under the museum model in the private domain [3] or under a 'boutique' model [4] in the public domain, whereby small teams curate organism- or process-specific datasets from the primary literature for short-term research purposes. Such decentralized resources are disseminated and maintained in *ad hoc *ways that are often not integrated with the major genome database resources, and can present a bewildering array of choices to the computational or experimental end-user.

Recently, two efforts have been launched to develop integrated portals for *cis*-regulatory annotation - ORegAnno [5] and PAZAR [4] - that aim to support research in *cis*-regulatory sequence and network analysis. Both ORegAnno and PAZAR provide principled, standardized technologies for the long-term, community-driven, open-access annotation of *cis*-regulatory data in the context of the major genome database resources (for example, National Center for Biotechnology Information (NCBI), Ensembl, University of California Santa Cruz (UCSC)) and, as such, represent a new generation of resources for the annotation of *cis*-regulatory data. Despite these advances in infrastructure, many challenges still remain for the comprehensive community-based annotation of *cis*-regulatory data. First, as with all decentralized annotation efforts, community annotation of regulatory data from the literature requires systems to track the curation process, including 'triaging' relevant and irrelevant articles and monitoring the curation status of papers. Second, the scale of the *cis*-regulatory annotation challenge remains unknown, and thus it is critical to identify and prioritize the set of documents with high *cis*-regulatory potential for curation. Third, with curation times currently on the order of approximately one to two hours per paper, a major bottleneck remains in how to efficiently extract *cis*-regulatory data from primary text. Recently, rule-based information extraction systems have been developed to extract regulatory relations among pairs of genes and proteins [6-8]; however, many other types of data are necessary for comprehensive *cis*-regulatory annotation, such as the organism under investigation and, perhaps most importantly, the sequence and genomic location of *cis*-regulatory elements.

We have attempted to solve some of these challenges through the use of text-mining techniques to retrieve and extract relevant documents and data for the annotation of *cis*-regulatory networks and sequences. These efforts were inspired by (and conducted in part through) the RegCreative Jamboree [9], a workshop that was held in late 2006 that attempted to explore the interface between regulatory bioinformatics and text-mining communities. Elsewhere [10], we detail the development of a literature management system for the regulatory annotation community, which warehouses the set of papers that are likely to contain *cis*-regulatory data and maintains information on their current curation status. Here we develop a vector space model to identify Medline abstracts of papers that are likely to have high *cis*-regulatory content, and use this model to demonstrate that document relevance ranking can assist the annotation of transcriptional regulatory networks and be used to estimate the scale of the regulatory curation challenge. In addition, we show that DNA sequences can be extracted from full-text articles and mapped to genome sequences as a means to identify the location, organism and target gene information that is critical to the *cis*-regulatory annotation process. Collectively, our results demonstrate the utility (and the necessity) of employing text-mining approaches to accelerate the community-driven annotation of *cis*-regulatory sequences and networks that control transcription.

## Results

### A literature management system for community annotation and text mining

Assembling the set of documents that are relevant for annotation and tracking the curatorial status of papers are major challenges in community annotation. To help overcome these issues, we have developed a literature management 'queue' for the ORegAnno database, which allows registered users to input papers with known or suspected *cis*-regulatory content as targets for curation using their PubMed identifiers (PMIDs). A full description of the ORegAnno Publication Queue and its features is detailed elsewhere [10]; here, we briefly describe its contents to aid interpretation of our text-mining results. The ORegAnno Publication Queue was initially populated with expert entries obtained from the set of papers in ORegAnno plus existing sources of curated publications, including the *Drosophila *DNase I Footprint Database [11], REDfly [12], a catalog of regulatory elements for muscle-specific regulation of transcription [13,14], ABS [15], TRED [16], ooTFD [17] and DBTGR [18]. Additionally, a large number of papers were added manually by individual ORegAnno users from literature searches and review articles. Together, these PMIDs form the 'expert entry' component of the ORegAnno Publication Queue. In the current work, we show how, in addition to offering a powerful literature management system for community annotation, the ORegAnno Publication Queue offers a rich source of PMIDs for assessing information retrieval and information extraction techniques applied to biomedical text in the *cis*-regulatory domain.

### A vector space model identifies Medline abstracts with high *cis*-regulatory content

**Figure 1:**
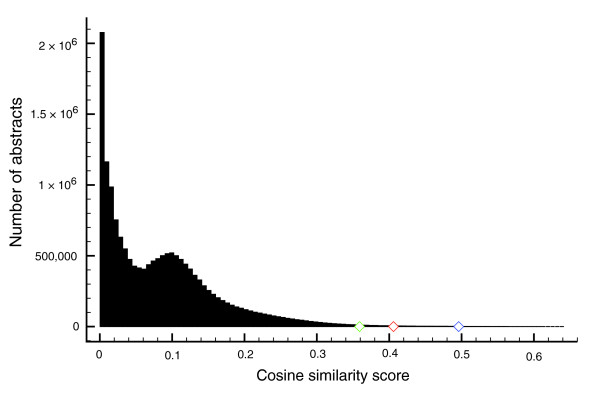
Distribution of cosine similarity scores between the query vector and each of the Medline abstract vectors, indicating the 10,000th (blue diamond) 50,000th (red diamond) and 100,000th (green diamond) ranked abstract.

As a first step in employing text-mining to aid *cis*-regulatory annotation, we attempted to identify a set of full-text papers that could enter the curation process by using information retrieval technology. To do this, we implemented a vector space model [19] that scores the approximately 16 million scientific abstracts from Medline, each represented as a vector of index terms, against a model trained on a corpus of abstracts that *a priori *are known to have high *cis*-regulatory content. For initial model training purposes, 3,626 abstracts retrieved with the Pubmed query 'transcription and regulation and 'binding site' and (promoter or enhancer)' (see Materials and methods for details) were first split into two equal parts that form a training set (*POS1*) and a validation set (*POS2*). *POS1 *contains 3,344 terms after stemming and stop-word removal, representing vocabulary *VOC1*. We compared ten different relevancy rankings with *POS1 *as query and either the complete *VOC1 *or different subsets of *VOC1 *as vocabulary. A vocabulary consisting of the 1,000 terms with the highest frequency in the full corpus yielded the highest performance when applied to *POS2 *(results not shown). Similar results were obtained using a training set of 6,306 abstracts from papers previously curated in ORegAnno [5], TRANSFAC [3], or FlyReg [11]. Thus, we chose to develop our relevance ranking based on our '*cis*-regulatory' PubMed query to avoid biases towards data type, species, or other unknown factors. This approach has the additional advantage that existing sets of curated papers can legitimately be used later as validation sets. To generate the final relevancy ranking of Medline used in further analyses we used a model based on the 1,000 terms (from the 3,626 training abstracts) with the highest corpus frequency as vocabulary. Figure 1 shows the distribution of the final similarity scores for all approximately 16 million abstracts in Medline, with an indication of the top 10,000, top 50,000 and top 100,000 highest scoring abstracts in the distribution (these lists are called top10k, top50k, top100k and so on throughout the following text).

Using a similarity-based ranking rather than a classification procedure is particularly useful for our task because it does not require a negative training set, and because a similarity score allows a prioritization of documents for curation rather than a binary decision. To evaluate whether our similarity-based ranking agrees with other information retrieval technologies, we classified the entire 16 million Medline abstracts using a support vector machine (SVM) [20,21] trained on the same set of papers from our initial PubMed query as positives, and an equivalent number of randomly selected Medline abstracts as negatives. Using a radial basis function kernel, we find that 169,402 (1.07%) Medline abstracts are classified as positive and 95.6% of the top100k abstracts identified by our cosine similarity method are called positive by the SVM approach. Cosine similarity values and SVM decision function values are, furthermore, highly correlated (Pearson correlation coefficient is 0.88); 78.4% of abstracts are shared by the top100k when ranked by their cosine or SVM scores. Therefore, the cosine similarity and SVM methods both point to a very large but similar set of abstracts in Medline as having high *cis*-regulatory potential.

The coverage of several validation sets within the final ranking is shown in Table 1. Before calculating the sensitivity (recall) for each validation set, we removed all Medline abstracts from these sets that were also part of the training set. As a first validation set we used TRANSFAC [3], a commercial database of manually curated transcription factor binding sites (TFBSs). We collected all 5,719 PMIDs from TRANSFAC (v10.4) that are linked to a curated TFBS. Of the set of 5,183 independent TRANSFAC PMIDs (536 were part of the training set), 75.4% are found within the top50k and 88.2% within the top100k abstracts. This shows that our model is able to generalize and recover many true positive abstracts with high *cis*-regulatory content. In fact, the vector space model realizes an increase in the proportion of TRANSFAC PMIDs from 14.7% in the 3,626 papers based on the initial PubMed query to 18.8% in the top 3,626 publications after relevancy ranking. Likewise, using a second validation set of 186 independent positive PMIDs from the FlyReg database of curated TFBSs in *Drosophila*, we find high sensitivities of 78.5% and 89.2% of FlyReg PMIDs in the top50k and top100k scoring abstracts in Medline, respectively.

**Table 1:** Coverage of validation sets (excluding PMIDs in the training set) within the top10k, top50k, and top100k ranked abstracts for the vector space model relevancy ranking

	TRANSFAC	FlyReg	ORegAnno Queue	ORegAnno prior to RegCreative	RegCreative success	RegCreative failure
Number of PMIDs	5,719	200	4,145	376	260	218
Number of PMIDs (no training data)	5,183	186	3,687	340	228	212
Number in top10k	1,390	38	1,035	89	59	18
Percent in top10k	26.8%	20.4%	28.1%	26.2%	25.9%	8.5%
Number in top50k	3,908	146	2,753	260	165	79
Percent in top50k	75.4%	78.5%	74.7%	76.5%	72.4%	37.3%
Number in top100k	4,572	166	3,208	301	199	110
Percent in top100k	88.2%	89.2%	87.0%	88.5%	87.3%	51.9%

Next, we investigated the coverage of true positive abstracts using curated papers from the ORegAnno database [5], including those curated as a part of the RegCreative Jamboree [9]. Prior to the Publication Queue, ORegAnno contained 376 curated papers, of which 340 are not part of the training set in the vector space model. Of these, 88.5% (n = 301) are covered in the top100k. Since the creation of the Publication Queue, curated papers are flagged with 'failure' or 'success,' depending on whether they contained enough data to allow the creation of a full ORegAnno record (that is, either a regulatory region or a TFBS with all required fields; see above). Surprisingly, in a set of 478 papers from the ORegAnno Publication Queue (see above) that were known *a priori *to have a high likelihood of containing curatable *cis*-regulatory data, only 54.4% (n = 260) were confirmed as 'success' papers during the RegCreative Jamboree. The remaining 218 'failure' papers contained either no regulatory data, or one or more critical data fields were missing (for example, the regulatory sequence could not be identified or unambiguously mapped to a target gene or species). Excluding training abstracts, 87.3% (n = 199) of the success papers are found in the top100k but only 51.9% (n = 110) of the failure papers are found in the top100k, indicating that our relevance ranking increases the likelihood that a paper has curatable *cis*-regulatory data. Collectively, these experiments show that our vector space model successfully identifies and ranks papers with enriched *cis*-regulatory content based on Medline abstracts, and that information retrieval techniques can be used to populate a larger ORegAnno Publication Queue to assist the community annotation of *cis*-regulatory data.

### Estimating the size of the *cis*-regulatory corpus

**Figure 2:**
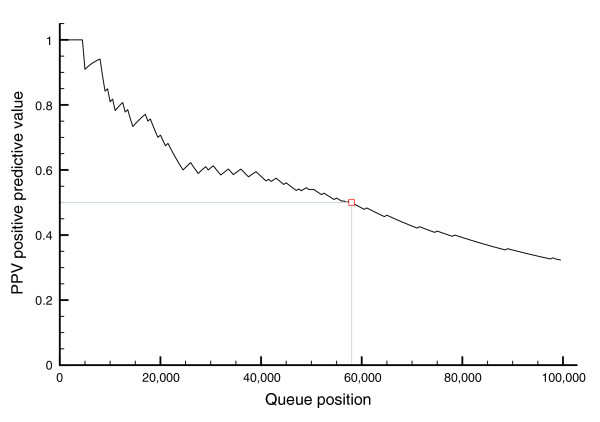
PPV calculated for each threshold in the top100k of the final relevancy ranking, using the pseudo-curation results of 200 evenly distributed samples. The length of the final 'text-mining entry' component of the ORegAnno Publication Queue was chosen at 58,000, which yields a PPV of 50%.

**Figure 3:**
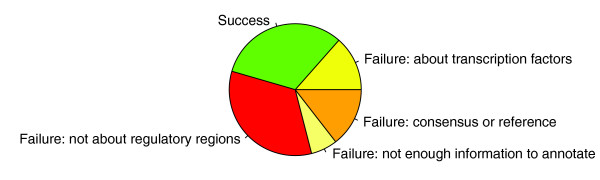
Results of the pseudo-curation procedure on 200 evenly distributed samples across the top100k.

Although the sensitivities of our vector space model on evaluation sets are high, the calculations were performed on large sets of PMIDs (10k, 50k or 100k), meaning that the majority of candidate papers do not fall into any of the existing sets of curated papers. To investigate the degree to which the additional predictions show high true positive rates, we conducted a validation experiment that also gives us an indication of the scale of the *cis*-regulatory annotation challenge. We constructed a sample of 200 PMIDs evenly spaced every 500 abstracts across the top100k abstracts. Full-text papers for these 200 samples were subjected to a 'pseudo-curation' procedure in which the paper was read by an expert and, instead of being fully curated, was only scored with respect to its 'curatability' for containing a TFBS (see Materials and methods). This experiment allowed us to estimate how the proportion of true positives and false positives vary as a function of position in the ranked list of the top100k scoring Medline abstracts. Figure 2 shows the positive predictive value (PPV) for each threshold of the top100k. The first 10 samples were all success papers, indicating that the top scoring 4,501 papers are extremely likely to contain curatable *cis*-regulatory data. From then onwards, the PPV starts to decrease but still remains above 30% for the entire top100k scoring abstracts. This curve can be used to determine an optimal threshold for including papers in the ranked Medline list into the ORegAnno Publication Queue. As noted above, the proportion of success papers from the expert-entry ORegAnno Publication Queue was 54.4% during the RegCreative Jamboree. To achieve a similar curation success rate in the set of papers identified by the vector space model (namely PPV approximately 50%), we would include the top 58,000 scoring abstracts. Therefore, we estimate that the scale of the full corpus with curatable *cis*-regulatory data in Medline is on the order of approximately 30,000 papers. We note that this is a conservative measure because the success criteria are strict. Indeed, among the failure papers are many that contain regulatory data or references to other potential success papers (Figure 3). Based on these results, we added PMIDs and ranks for the top 58,000 scoring papers in Medline as 'text-mining entries' to the ORegAnno Publication Queue.

### Abstract relevance ranking aids the construction of regulatory networks

**Figure 4:**
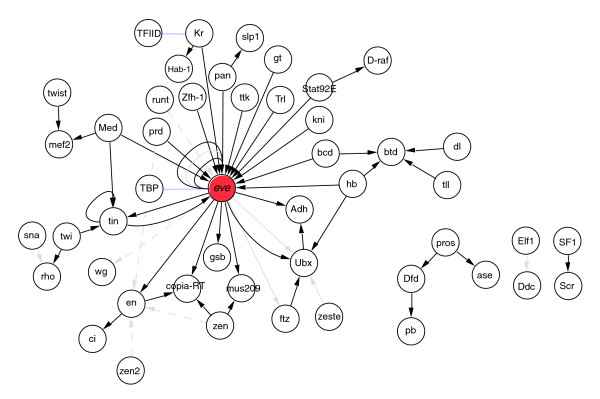
Transcriptional regulatory sub-network around the *Drosophila *transcription factor *even-skipped *(*eve*). All nodes and edges were retrieved from *eve*-related publications in the top100k abstract list. Black edges are success papers (that is, fully curatable publications); grey edges are failure papers that report regulatory data (for example, consensus sites) but are not the primary reference; grey dashed edges are failure papers that contain regulatory data that are not complete enough to allow full curation; blue edges are failures that report protein-protein interactions.

To illustrate the utility of identifying papers with high *cis*-regulatory content, we queried the top58k scoring abstracts for a particular transcription factor (TF), namely the *Drosophila *homeodomain-containing gene *even*-*skipped *(*eve*). Our goal was to use the set of papers enriched for *cis*-regulatory content to construct a literature-based transcriptional regulatory network focused on the upstream regulating factors and downstream target genes (TGs) of *eve*, based on high-quality published TFBS data. For this experiment we started with the entire list of 664 references associated with *eve *in FlyBase [22], which also includes papers not related to *cis*-regulatory data (for example, genetic interactions). We cross-referenced this list of all papers on *eve *with the top58k list to filter for papers on *eve *that are likely to contain *cis*-regulatory data. Of the 664 *eve *papers, 88 are found in the top58k list (147 are in the top100k), and for 85 of those (144 for the top100k) we retrieved the full PDF paper. We conducted a pseudo-curation analysis on these 85 papers to identify those that reported binary TF→TG relationships. We classified 35 out of these 85 candidates as 'success' papers, which revealed 43 unique binary TF→TG relationships (there were 47 relationships in total, including 4 relationships that occurred twice), 20 of which involved *eve *either as TF or as TG. A summary of the identified regulatory interactions is presented in Figure 4 as a network constructed using Cytoscape [23]. By comparison with previously curated binary TF→TG relationships for *eve *in the FlyReg database [11], our automated document retrieval process recovered 100% (12 of 12) of known upstream activating TFs, and 85% (6 of 7) of known downstream TGs. The only downstream TG curated in FlyReg that was missing in this analysis was *Abdominal-A *(*Abd-A*), which was omitted because it was not present in the original list of *eve*-related papers curated by FlyBase. These results show that cross-referencing general PMID lists for a given gene against our vector space model can enrich for papers that report direct *cis*-regulatory interactions for that gene, that transcriptional regulatory networks can be assembled from text-extracted binary TF→TG relationships [6-8,24], and that TF→TG interactions may be extracted from text even when full curation of *cis*-regulatory sequences may not be possible.

### Full-text articles contain *cis*-regulatory sequences that can be automatically mapped to genomes

We also evaluated the possibility of automatically annotating *cis*-regulatory sequences from publications with high *cis*-regulatory content by extracting DNA-like strings from text and mapping these putative DNA sequences to genomes. Previously, it has been shown that short protein and nucleic acid sequence strings can be extracted from text with high precision, and that many extracted DNA sequences correspond to regulatory sequences or motifs [25]. Using automated downloads of full-text articles based on the NCBI eutils, followed by HTML-scanning for links that end with 'pdf,' we obtained PDFs for 86.9% (n = 9,940) of 11,437 papers with high *cis*-regulatory content. This recovery rate of PDFs from PMID lists is slightly higher than a rate of 79.6% reported for papers on bacterial gene regulation [8]. We converted 95.0% (9,440/9,940) of full-text PDFs into plain text files of greater than 2,000 bytes, a cutoff that represented the lower size of converted files with *cis*-regulatory content based on manual inspection. We extracted DNA-like strings from 85.4% (8,066/9,440) of these text files using a rule-based approach involving regular expressions and word size cutoffs (see Materials and methods). In total, we obtained nearly 2.8 Mb of DNA-like text from these 8,066 papers. We obtained BLAST hits of 10e-5 or greater to at least one of the five genomes under investigation for DNA sequences from 36.9% (2,975/8,066) of the PMIDs with extractable fasta sequence. Numbers of documents obtained at each stage of the process for the different source PMID lists are shown in Table 2. Overall, the proportion of papers with sequences that can be mapped to one of the five genomes is 26.0% (2,975/11,437), with the lowest efficiency step being the mapping of short sequence elements to genomes. Similar results were obtained using a previously reported Markov chain method [25] to extract DNA sequences from full-text (data not shown), with differences mainly attributable to the inclusion of lowercase DNA characters by the method of Wren *et al. *[25].

**Table 2:** Efficiency of document recovery, sequence extraction and genome mapping for the source lists of PMIDs with high *cis*-regulatory content

	TRANSFAC	FlyReg	ORegAnno	Queue	top4,501	All
Number of PMIDs	5,719	202	914	4,145	4,491	11,437
Number of PMIDs with PDF	5,302	187	835	3,710	3,677	9,940
Percent PMIDs with PDF	92.7%	92.6%	91.4%	89.5%	81.9%	86.9%
Number of PMIDs with text >2 Kbytes	5,051	175	793	3,517	3,498	9,440
Percent PMIDs with text >2 Kbytes	88.3%	86.6%	86.8%	84.8%	77.9%	82.5%
Efficiency of text conversion	95.3%	93.6%	95.0%	94.8%	95.1%	95.0%
Number of PMIDs with fasta sequence	4,357	155	660	3,044	3,080	8,066
Percent PMIDs with fasta sequence	76.2%	76.7%	72.2%	73.4%	68.6%	70.5%
Efficiency of sequence extraction	86.3%	88.6%	83.2%	86.6%	88.1%	85.4%
Number of PMIDs with fasta sequence mapped to genome	1,518	75	303	1,279	1,260	2,975
Percent PMIDs with fasta sequence mapped to genome	26.5%	37.1%	33.2%	30.9%	28.1%	26.0%
Efficiency of genome mapping	34.8%	48.4%	45.9%	42.0%	40.9%	36.9%

Note that totals are less than the sum of the sets since many PMIDs are found in more than one source list.

**Figure 5:**
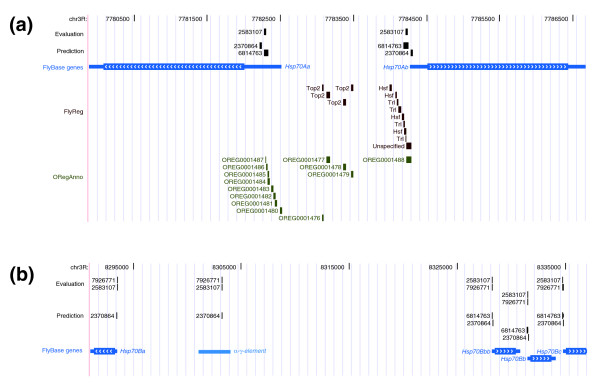
Comparison of automatically extracted text-based annotation and manual annotation of the *D. melanogaster Hsp70 *gene regions. **(a) **The *Hsp70Aa-Ab *region. **(b) **The *Hsp70Ba-Bc *region. The 'evaluation' track refers to text-based hits extracted from papers with curated regulatory data in ORegAnno; the 'prediction' track refers to text hits extracted from papers not currently curated in ORegAnno, but with high predicted *cis*-regulatory content. Annotations in both text-based tracks are labeled with their corresponding PMIDs. Also shown are the original manual annotation in the FlyReg database, the automated mapping of these curated data in ORegAnno, and FlyBase genes, including the *α-γ-element *noncoding RNA gene that is expressed in response to heat shock. Differences in the FlyReg and ORegAnno mappings in (a) arise because the sequences for these regions are duplicated in the genome and alternative unique mappings are chosen in the two databases.

To provide biologically meaningful *cis*-regulatory annotations, automatic text-based sequence extraction must identify genomic regions that match true *cis*-regulatory elements but not a large number of other irrelevant features. To test this we used a set of 3,208 regulatory elements with known genomic location from a list of 850 'evaluation' papers with manually curated entries in ORegAnno. Three papers (PMIDs 12566409 [26], 17086198 [27] and 17558387 [28]) with 947 ORegAnno records from high-throughput experiments in humans that were imported in bulk into ORegAnno were omitted from this analysis. The numbers of regulatory elements annotated in ORegAnno, regions mapped with extracted text, and their overlap are shown in Table 3. Overall, the PPV of our approach is reasonably high (64.8%), typically with lower PPV in large mammalian genomes (42.2-70.6%) and higher PPV in small invertebrate genomes (79.3-81.3%). At the *cis*-regulatory element level, sequences overlapping approximately 33% of known ORegAnno annotations overall can be obtained directly from primary text and mapped to genomes. For *Drosophila melanogaster*, we find that text-based regulatory sequence extraction can yield annotations that have a higher PPV but lower sensitivity than the best *de novo *regulatory element prediction methods [29]. Higher sensitivities for text-based regulatory sequence prediction are observed in mouse and rat (58.4-59.8%) relative to human, worms and flies (12.4-32.8%), which can be explained by the fact that these latter species have been the subject of dedicated annotation efforts in ORegAnno and are likely to contain a deeper level of human inference in their annotation. Since only 54.4% of papers were deemed 'success' papers in the RegCreative Jamboree (see above), these relatively low sensitivities are perhaps not surprising and indicate that, in some species, we may be achieving sensitivities approaching the upper bound of what is possible automatically. An example of the accuracy and utility of text-based regulatory sequence extraction is shown in Figure 5. The *Hsp70 *promoter region is duplicated seven times in the *D. melanogaster *genome, with only one locus currently annotated in FlyReg (*Hsp70Ab*). Our method cleanly extracts and correctly maps several *Hsp70 *regulatory elements from full-text to genome coordinates, both from previously annotated ('evaluation') papers plus other ('prediction') papers not currently annotated in ORegAnno (Figure 5a). In addition, the unbiased nature of our method improves the current annotation of *Hsp70 *regulatory sequences in *Drosophila*, with text hits mapping to all six copies of the *Hsp70 *gene as well as the promoter region of the *α-γ-element *noncoding RNA gene that is expressed in response to heat shock [30,31] (Figure 5a,b).

**Table 3:** Performance of text-based sequence extraction for *cis*-regulatory annotation

	dm2	hg18	mm8	ce2	rn4	All
Number of ORegAnno annotations	2,079	589	255	178	107	3,208
Number of PMIDs with ORegAnno annotation	389	283	113	30	48	850
Number of PMIDs with Ensembl target gene name(s)	388	253	107	29	42	819
Number of text hits from PMIDs with ORegAnno annotation	188	128	51	16	32	415
Number of text hits that overlap ORegAnno annotation	149	54	36	13	17	269
Percent text hits that overlap ORegAnno annotation (PPV)	79.3%	42.2%	70.6%	81.3%	53.1%	64.8%
Number of ORegAnno annotations overlapped by a text hits	681	133	149	22	64	1,049
Percent ORegAnno annotations overlapped by a text hits (SN)	32.8%	22.6%	58.4%	12.4%	59.8%	32.7%
Number of PMIDs with text hits	124	91	44	12	24	295
Percent PMIDs with text hits (coverage)	31.9%	32.2%	38.9%	40.0%	50.0%	32.2%
Number of PMIDs with text hits to correct species	123	84	37	12	18	274
Percent PMIDs with text hits to correct species (PPV)	99.2%	92.3%	84.1%	100.0%	75.0%	92.9%
Number of PMIDs with text hits and Ensembl target gene name(s)	122	77	33	11	16	259
Number of PMIDs with text hits and perfect match to correct target gene name(s)	67	57	24	4	10	162
Number of PMIDs with text hits and partial match to correct target gene name(s)	16	12	5	3	4	40
Percent PMIDs with text hits and match to correct target gene name (PPV)	68.0%	89.6%	87.9%	63.6%	87.5%	78.0%
Number of PMIDs without ORegAnno annotation with text hits	76	1,291	841	13	459	2,680
Number of text hits from PMIDs without ORegAnno annotation	126	2,602	2,131	14	1,002	5,875
Number of text hits from PMIDs without ORegAnno annotation that overlap ORegAnno annotation	59	202	58	1	18	338
Number of ORegAnno annotations overlapped by text hits from PMIDs without ORegAnno annotation	200	347	139	3	33	722

### DNA sequences extracted from text identify organisms and target genes

The organism referred to in a paper critically affects systems that attempt to recognize gene names in biomedical text and cross-reference them to external database identifiers [32]. Species identifiers are also a mandatory field in the ORegAnno curation process. Thus, we investigated if our sequence extraction and genome mapping process may provide a novel solution to the species identification problem in text mining. Of the 850 unique PMIDs with ORegAnno annotations in one or more of five species studied here (11 PMIDs have ORegAnno records for 2 species, and 1 PMID has ORegAnno records for 3 species), 295 had best genome hits obtained from extracted sequences. The correct species was identified using the genome with highest scoring BLAST hit for 92.9% (274/295) of PMIDs with hits extracted from text and ORegAnno annotations. We manually inspected the best genome hits that were incorrectly assigned to the wrong species and found that the vast majority were for hits among the three closely related mammalian species studied here (rat, mouse and human). Most of these incorrect assignments result from the requirement of a single best genome match, which can cause the wrong species identification for two reasons: first, a single PMID may report sequences (and therefore have ORegAnno records) for multiple species but only a single species gets chosen; second, only a single species is reported in the paper and annotated in ORegAnno, but the wrong species is assigned because the sequences (and BLAST scores) in another species are identical. In addition, a small number of 'incorrect' species assignments are because the species was actually incorrectly curated in the current ORegAnno annotation (for example, OREG0000115). These incorrect annotations have been deprecated and replaced by correct annotations in ORegAnno (for example, OREG0004685). These results demonstrate that primary text contains valuable information about the species under investigation encoded in extractable DNA sequences, but that mistaken species assignments may occur among closely related species or when sequences from multiple species are reported in a single paper.

Gene name recognition and normalization to database identifiers is an essential step in many text mining applications, but is a challenging task because of ambiguity and variation in how genes are named and used [33]. The identity of the target gene regulated by a *cis*-regulatory sequence is a key piece of information in regulatory bioinformatics and is a required field in an ORegAnno annotation. Thus, we investigated whether it is possible to automatically identify the target gene of putative *cis*-regulatory sequences extracted from text and mapped to genomes. To do this we simply identified the closest Ensembl gene to each text hit that was mapped to one of the five genomes. In the case of text hits found in introns, the closest gene was predicted to be the gene containing the intron, even if additional genes were present within the intron that were closer to the text hit. Each hit for PMIDs that generated multiple genomic hits was assigned its own putative target gene and evaluated for whether any of the PMID-target gene relationships were found in ORegAnno. For this analysis, we used a set of 259 PMIDs with ORegAnno annotations that provided a best hit to one of the five genomes and for which one or more predicted target gene names were found in the set of Ensembl normalized GeneIds in ORegAnno. For 162 PMIDs, the list of closest genes matched the list of correct target genes perfectly, and for an additional 40 PMIDs there was a partial match between the list of putative target genes and the true list of target GeneIDs in ORegAnno. Overall, 78.0% of PMIDs generated at least one text hit whose closest gene was the correct target gene. In general, extracting sequences from text yields a higher proportion of correct target genes (87.5-89.6%) in the larger mammalian genomes where gene density is relatively low. In contrast, in the compact genomes of *D. melanogaster *and *Caenorhabditis elegans*, a lower proportion of target genes is correctly identified (63.6-68.0%) since a text hit can have a higher probability of being closer to a neighboring gene than its true target in a compact genome. Remarkably, our simple DNA sequence-based gene name recognition method achieves levels of PPV (precision) that are higher than the median performance in BioCreAtIvE Task 1B [32] of advanced gene name recognition systems for flies (65.9%) and mice (76.5%). Additionally, since each PMID with a text extracted hit leads to at least one predicted target gene, our sequence extraction method identifies gene names from full-text articles at a rate (26.0%) comparable to dictionary-based gene name recognition in Medline abstracts (19.4%) [34].

### A draft annotation of more than 2,000 papers with high *cis*-regulatory content

Among the 10,587 papers not currently curated in ORegAnno in our set of 11,437 PMIDs with high *cis*-regulatory content, we obtained hits to 5,875 genomic regions from 2,680 PMIDs. If we assume that approximately 65% of text hits from these 'prediction' papers are true positives (based on the overall PPV estimates above), we expect that approximately 3,800 of these text hits correspond to *cis*-regulatory sequences. The addition of these records would increase the number of annotations curated from small-scale experiments in ORegAnno by approximately 120%. Indeed, many of these are likely to be *bona fide *regulatory sequences, as shown by the fact that 338 text hits from papers not currently curated overlap 722 pre-existing ORegAnno annotations. For example, PMIDs 6814763 [35] and 2370864 [36] (which were both identified as having high *cis*-regulatory content by our vector space model) each provided an extractable sequence that mapped to previously annotated *cis*-regulatory elements in the *Hsp70 *promoter (Figure 5a). This result suggests even the most highly curated genomes have yet to achieve 'saturation annotation' and that a high level of redundant publication may exist for some regulatory elements, which can be used to support or extend current ORegAnno annotations. These predictions are not sufficient to stand as full ORegAnno records on their own, but should substantially decrease the time needed for the community annotation of these papers. In addition, these regions may be of sufficient resolution to be used by other workers in regulatory bioinformatics, and for these reasons we provide browser extensible data (BED) files for text-extracted sequences from both evaluation and prediction papers for the *D. melanogaster *(Additional data file 1), human (Additional data file 2), mouse (Additional data file 3), *C. elegans *(Additional data file 4), and rat (Additional data file 5) genomes.

## Discussion

A principle aim of genome biology is to decode complete transcriptional networks, so as to better understand how the activation of specific subnetworks affect developmental processes or responses to the environment, and how variation in transcriptional networks can lead to functional diversity over evolutionary time. As with all grand challenges in interpreting genome sequences, solving this ultimate aim will require combining both computational and experimental approaches. As the reliability of predictive regulatory sequence bioinformatics is relatively low [37], high-throughput experimental techniques currently prove to be the most efficient means of identifying regulatory sequences and assembling regulatory networks [38,39]. The gold standard for evaluating both computational and high-throughput experimental techniques continues to be the sizable body of prior knowledge contained in small-scale experimental studies on *cis*-regulatory sequences, much of which remains locked in the biomedical literature. Here we have shown that application of text-mining technologies, including literature management, information retrieval and information extraction systems, can accelerate the community annotation of *cis*-regulatory networks and sequences. These advances should help generate the necessary training and test sets to improve the reliability of computational and high-throughput experimental methods in regulatory biology.

Previously, it has been shown that manually curated and automatically extracted binary TF→TG interactions can be assembled into transcriptional regulatory networks [6-8,24]. Here we show that abstract relevance ranking using a vector space model can be used to enhance the manual annotation of binary TF→TG interactions, and should likewise further improve the automated extraction of binary TF→TG interactions to construct regulatory networks. We have also shown that the binary TF→TG interactions that are central to the construction of transcriptional regulatory networks can be extracted from text even when a full curation of the *cis*-regulatory sequence responsible for this interaction may not be possible. Our vector space model also has allowed us to generate an enhanced 'queue' of papers for annotation, and to gain a deeper insight into the size of the corpus of papers that may contain curatable *cis*-regulatory sequences, which we estimate is on the order of 30,000 papers or more. At the rate of approximately 1-2 hours curation time per paper, it would take a single person approximately 15-30 years to curate and annotate this corpus manually. This estimate demonstrates the need for distributed community annotation systems and for computational tools that can assist the extraction of relevant *cis*-regulatory information.

We have also investigated the potential of exploiting information contained in the DNA sequences reported in papers with high *cis*-regulatory content to assist regulatory annotation. Given the large number of DNA, RNA and peptide sequences reported in the biomedical literature, and the fact that sequences important enough to deserve mention in publication are likely to be of high biological significance, surprisingly little work has been conducted on extracting sequences from primary text [25,40]. The pioneering work of Wren *et al. *[25] showed that Markov models trained on English text, proteins and/or genomic DNA can be used to extract both DNA and peptide sequences from abstracts and full text with high precision. Wren *et al. *[25] also demonstrated that the extraction of DNA is more precise than peptides, and that the terminological context of the majority of extracted DNA sequences revealed that the sequence was likely to be a 'regulatory site' or 'motif' [25]. Our results directly support the claim that primary text contains a large number of DNA strings that are *cis*-regulatory sequences, which we also show can be automatically mapped to genome sequences to accelerate and enhance regulatory annotation. In addition to validating our approach, overlaps between ORegAnno annotations and text-based hits can be used as an automatic procedure to authenticate ORegAnno annotations, which can be indicated in the 'Score' profile for each ORegAnno record. As identifying and annotating *cis*-regulatory sequences in genomes currently remain among the most challenging branches of bioinformatics, ironically it may now be easier and more productive to identify functional *cis*-regulatory sequences in biomedical text rather than in DNA itself.

Our rule-based system for extracting and mapping DNA sequences could potentially be improved in several ways. One area to explore would be to implement more sophisticated sequence recognition techniques such as Markov models [25], although our initial comparisons suggest very similar overall performance. Inclusion of lowercase letters or degeneracy in the DNA alphabet of our rule-based method may allow many more *cis*-regulatory motifs to be extracted, but may also allow many more DNA-like English words to be extracted. Aside from variation in formatting [25], DNA strings in text should be easily discernable from English words and, therefore, identifiable by many alternative methods, since the upper limit of English words that can be spelled entirely in the DNA alphabet is small. For example, in a dictionary of approximately 355,000 English words [41], only 47 can be spelled entirely in DNA letters [ACGT], with an upper length of 7 characters for the word 'attacca,' a directive used at the end of a piece of music that is unlikely to be found in biomedical text. Inclusion of the entire set of ambiguity codes for DNA [ACGTMRWSYKVHDBXN] leads to a maximal English word size of only 13 characters for 'dharmashastra,' an ancient form of Indian jurisprudence. Thus, the vast majority of DNA-like strings of sufficient length to be mapped unambiguously to genomes are almost certainly *bona fide *DNA sequences. The main challenge for extracting DNA from text will be inaccuracies in the text encoding in older PDF documents, and the fact that many DNA sequences are embedded in tables, figures and supplementary materials. Although some figures have corresponding text encoded in the PDF, the use of text-recognition algorithms that operate on images would almost certainly improve the predictive power of our approach, and preliminary experiments have shown that this is the case (results not shown).

The area with the largest scope for improvement in using DNA in text to annotate genomes is the mapping of sequences to genomes (Table 2), in part because of the short length of many *cis*-regulatory sequences. One way to solve this problem would be to combine sequence extraction with term recognition [25] to identify species or target gene names that could be used to reduce the search space for mapping extracted sequences to genomes. Another improvement would be to accept mappings to multiple species, which is also a more realistic solution than the requirement for a single 'best' species since the biological function of a reported sequence is likely to be the same closely related species. Improvements may also come from more lenient BLAST thresholds or the use of non-RepeatMasked versions of genomes, although these would almost certainly lead to higher false positive rates. Mapping regulatory sequences to repetitive genomic regions is a general problem, not only for text-extracted sequences, but also for manually curated data (Figure 5a). However, since many *cis*-regulatory elements may arise from transposable element sequences [42] or be located in segmental duplications (Figure 5), it will be necessary to solve the problem of representing and storing repetitive *cis*-regulatory elements for comprehensive regulatory annotation.

As presaged by Lincoln Stein [1], our results demonstrate that it is indeed possible to leverage text-mining technologies to accelerate genome annotation. Our proof of principle in the field of regulatory annotation is only one potential application of text-based genome sequence annotation. The general combining of information retrieval systems (for example, [19]) with sequence extraction techniques (for example, [25]) should allow researchers to enrich for any specific sub-domain of biomedical research and use sequence data reported in these corpora to directly annotate genomic regions of interest in a highly automated fashion. For example, the false positive mappings that correspond to coding sequences in our set of documents with high *cis*-regulatory content (see above) are likely to be mainly for proteins that bind to *cis*-regulatory sequences, and thus strategies similar to ours could accelerate the labor intensive identification of sequence specific TFs [43,44]. Clearly, it is preferable that researchers deposit and store their sequences and annotations in databases as a condition for publication and thereby preclude the need for post-publication extraction of such valuable biological data. With established databases for general sequence submission (for example, [45]) and specialized *cis*-regulatory annotation [4,5], researchers now have the necessary tools to deposit and archive their *cis*-regulatory data. In the absence of direct database submission, we recommend that researchers report certain minimum information (that is, absolute coordinates with genome build, sequence with sufficient flank, standard gene identifiers, official species name or identifiers) to assist the regulatory annotation (both human and automated) that is needed to help catalyze advances in the field of gene regulation.

## Materials and methods

### Implementation of a vector space model to identify Medline abstracts with high *cis*-regulatory content

To identify papers with potential *cis*-regulatory data for community annotation, we used a vector space model [19] that represents each of the approximately 16 million scientific abstracts in Medline as a vector of index terms. Each vector element is a weight that is proportional to the relative importance of the term in the abstract (using the inverse document frequency or IDF). Relevancy ranking of the corpus is then achieved by calculating the similarity between each abstract and a query. This query can be represented by the same kind of vector as the documents, so that the similarities can be calculated by the cosine similarity measure between individual abstract vectors and the composite query vector. In practice, a good query vector can be constructed from the average properties of a training set of true positive abstracts. In this study, we used a '*cis*-regulatory' PubMed query that yielded a very high amount of true positives to generate our training set, namely: 'transcription and regulation and 'binding site' and (promoter or enhancer)'.

### Pseudocuration of full-text articles

To evaluate the ability of our model to predict papers with high *cis*-regulatory content, we selected 344 papers from the top 100,000 scoring abstracts, of which 200 are uniformly distributed and 144 are related to the *Drosophila *transcription factor *eve*. Because the full curation of all 344 papers would require the organization of a second annotation jamboree, we opted for a distributed 'pseudocuration' procedure. Particularly, nine experienced curators examined whether these papers describe experimentally verified regulatory data and, if so, whether they also contain all the required data to allow genome annotation (that is, at a minimum the species, the sequence and its genomic location, the TF, and the TG). A web application was created where the curators could open a pending PMID and score the full-text paper as success or failure. Failures could be of four types: the publication describes binding site or promoter but there is insufficient information to annotate it; the publication describes transcription factor (complex) but not a binding site or promoter; the publication describes consensus binding sites or a reference to a primary publication but is itself not the correct source for annotation; and the publication does not describe a regulatory element. Regulatory interactions in the form of TF→TG were recorded as free text.

### Extraction of DNA sequences from full-text and mapping to genome sequences

A unique list of 11,437 PMIDs was compiled from papers previously curated in FlyReg [11], ORegAnno [5] TRANSFAC v10.4 [3], plus unannotated papers in the ORegAnno Publication Queue, and the top 4,501 scoring abstracts identified by the vector space model that are extremely likely to contain *cis*-regulatory data (see above). To allow access to information in both older and more recent articles, full-text was downloaded automatically as PDFs where available using a custom script employing NCBI eutils [46]. PDFs were converted to plain text using pdftotext (v3.0) with option '-nopgbrk' [47]. Text was split into words and words greater than 10 characters in length with greater than 40% of characters from the capitalized DNA alphabet [ACGT] were extracted using regular expressions to isolate putative DNA sequences. All putative DNA sequences extracted from each paper were concatenated in the order they appeared in the text into a single fasta sequence and labeled with the corresponding PMID. Concatenation of sequences was performed to merge sequences split by line breaks in the text conversion, and because we reasoned that inappropriate joins would be reconciled at the genome level by local alignment procedures. Extracted, concatenated sequences were used as queries to BLAST RepeatMasked versions of genome sequences downloaded from the UCSC genome database [48] for the five species with greater than 100 ORegAnno database annotations: *D. melanogaster *(dm2), human (hg18), mouse (mm8), *C. elegans *(ce2) and rat (rn4). We note that these five genomes represent approximately 99% of the records currently in ORegAnno. NCBI-BLASTN v2.2.10 [49] was used to map extracted sequences to genome coordinates with an E-value cutoff of 10e-5. BLAST output was parsed into BED format using Jim Kent's source tree utilities, blastToPsl and pslToBed [50]. BLAST results for all five species were concurrently searched to find the genome that provided the best sum of BLAST scores to each fasta sequence, and this list of PMID-best genome matches was used to filter BED files to minimize spurious cross-species mapping. We then joined fragmented hits in the same genomic interval by clustering BED annotations for the same PMID within 1.0 KB on the same chromosome. Filtered, clustered BED annotations were assessed for their overlap with the 20-JUL-2007 mapping of ORegAnno annotations [51] using the Kent source tree utilities overlapSelect and bedIntersect. Finally, we identified a single putative target gene for each hit as the Ensembl [52] GeneId closest to each filtered, clustered BED annotation.

## Abbreviations

BED, browser extensible data; NCBI, National Center for Biotechnology Information; PMID, PubMed Identifier; PPV, positive predictive value; SVM, support vector machine; TF, transcription factor; TFBS, transcription factor binding site; TG, target gene; UCSC, University of California Santa Cruz.

## Authors' contributions

SA, MH, SvV and CMB conceived of the study and conducted the text mining experiments and analysis. SA, OLG, SJMJ, SBM and CMB designed and implemented the ORegAnno Publication Queue. SA, MH, OLG, PH, SJMJ, SBM, CMB and The Open Regulatory Annotation Consortium contributed to the curation activities of the RegCreative Jamboree. SA and CMB drafted the manuscript and all authors read and contributed to the final manuscript.

## Additional data files

The following additional data files are available. Each additional data file is a UCSC genome BED formatted file that lists the chromosome, start coordinate, stop coordinate and PubMed identifier of text-extracted sequences on UCSC genome browser assemblies. Additional data file 1 provides genomic coordinates of text hits to the dm2 version of the *D. melanogaster *genome. Additional data file 2 provides genomic coordinates of text hits to the hg18 version of the human genome. Additional data file 3 provides genomic coordinates of text hits to the mm8 version of the mouse genome. Additional data file 4 provides genomic coordinates of text hits to the ce2 version of the *C. elegans *genome. Additional data file 5 provides genomic coordinates of text hits to the rn4 version of the rat genome.

## Supplementary Material

Additional data file 1 The UCSC genome BED formatted file lists the chromosome, start coordinate, stop coordinate and PubMed identifier of text-extracted sequences on UCSC genome browser assemblies. Click here for file

Additional data file 2 The UCSC genome BED formatted file lists the chromosome, start coordinate, stop coordinate and PubMed identifier of text-extracted sequences on UCSC genome browser assemblies. Click here for file

Additional data file 3 The UCSC genome BED formatted file lists the chromosome, start coordinate, stop coordinate and PubMed identifier of text-extracted sequences on UCSC genome browser assemblies. Click here for file

Additional data file 4 The UCSC genome BED formatted file lists the chromosome, start coordinate, stop coordinate and PubMed identifier of text-extracted sequences on UCSC genome browser assemblies. Click here for file

Additional data file 5 The UCSC genome BED formatted file lists the chromosome, start coordinate, stop coordinate and PubMed identifier of text-extracted sequences on UCSC genome browser assemblies. Click here for file
